# Smoking, alcohol consumption, diet and physical activity following stoma formation surgery, stoma-related concerns, and desire for lifestyle advice: a United Kingdom survey

**DOI:** 10.1186/s12889-019-6913-z

**Published:** 2019-05-15

**Authors:** Rebecca J. Beeken, Joanne S. Haviland, Claire Taylor, Anna Campbell, Abigail Fisher, Chloe Grimmett, Gozde Ozakinci, Sarah Slater, Iseult Wilson, Gill Hubbard

**Affiliations:** 1 Leeds Institute of Health Sciences, University of Leeds, Leeds, UK; 2 Institute of Cancer Research Clinical Trials and Statistics Unit, Division of Clinical Studies, The Institute of Cancer Research, London, UK; 3 St Mark’s Hospital, London North West NHS Healthcare, Harrow, UK; 4 Edinburgh Napier University, Edinburgh, UK; 5 Research Department of Behavioural Science and Health, University College London, London, UK; 6 University of Southampton, Southampton, UK; 7 School of Medicine, University of St Andrews, St Andrews, Scotland, UK; 8 Beatson West of Scotland Cancer Centre, Glasgow, Scotland, UK; 9 School of Nursing and Midwifery, Queen’s University Belfast, Belfast, UK; 10 Department of Nursing, University of the Highlands and Islands, Inverness, UK

**Keywords:** Stoma, Lifestyle, Physical activity, Diet

## Abstract

**Background:**

Adherence to smoking, alcohol consumption, diet and physical activity (PA) guidelines may improve outcomes for people with a stoma. A better understanding of these behaviours following stoma formation surgery and their experiences and attitudes towards receiving lifestyle advice, could help identify specific gaps and inform interventions going forward. The aim of this study was to describe changes in current lifestyle following stoma formation and to explore concerns, desire for lifestyle information, advice and support among people who have or have had a stoma.

**Methods:**

A sample of adults who currently had or in the past had a stoma for treatment for any medical condition was recruited online through relevant charities and companies, and invited to complete a cross-sectional, online survey. Consenting participants (*n* = 425) provided demographic information and completed brief, validated questionnaires about their lifestyle, alongside questions around their concerns regarding permanent stoma and experiences of lifestyle information and advice. Responses were summarised using descriptive statistics, and associations between reported concerns about stoma and changes in health behaviours were explored.

**Results:**

Most respondents (93%) still had a stoma at the time of completing the survey. The majority (80%) had not consumed at least 5 portions of fruit and vegetables on the previous day and 20% reported they had not participated in at least 30 min of physical activity on any day in the previous week. Most respondents were non-smokers (84%) and did not exceed recommendations for alcohol intake (60%). Most (56%) felt their PA had decreased following stoma formation. Frequencies of concerns about a permanent stoma were high, and appeared to be associated with reported decreases in PA. Of those reporting nausea, 40% felt their diet had worsened since having their stoma. A large proportion of respondents had not received PA (42%) or dietary (30%) advice, and of these > 90% would have liked guidance.

**Conclusions:**

Few respondents to this survey were eating the recommended amount of fruit and vegetables, and most reported a decrease in their PA following stoma surgery. Lifestyle advice would be welcomed by this population, which professionals should take into account when addressing stoma- related concerns.

**Electronic supplementary material:**

The online version of this article (10.1186/s12889-019-6913-z) contains supplementary material, which is available to authorized users.

## Background

A stoma is an artificial opening on the surface of the abdomen that has been surgically created in order to divert the flow of faeces or urine [1]. The three types of eliminating stomas are colostomy, ileostomy and urostomy, which can be temporary or permanent [1]. There are a number of conditions that may necessitate the formation of a stoma including colorectal cancer, diverticular disease, incontinence, and inflammatory bowel disease [1]. In the United Kingdom (UK), there are ~ 102,000 ostomates and around 21,000 stoma formation surgeries are performed annually [2]. Recent systematic reviews suggest that a stoma has a negative impact on quality of life (QOL) [3–5], and patients with a stoma report difficulties in work and social situations, with sexuality and body image, and with stoma function [6, 7]. In addition, complications are common and can exacerbate quality of life issues [8]. Interventions are therefore needed that have the potential to both reduce the risk of complication, and improve QOL for this group of patients.

A number of studies have demonstrated that lifestyle may be an important target to improve outcomes for people with a stoma. Lifestyle factors, such as diet, physical activity (PA) and smoking, have been associated with stoma-related complications [9–13], and are considered important for overall health, well-being, and mortality [14]. However, studies with colorectal cancer survivors suggest that the presence of a stoma is associated with a decrease in PA after diagnosis [15], and with less favourable lifestyle behaviours [16]. Similarly, a recent survey of 2631 people with a stoma found reductions in PA following stoma formation [17], but did not explore whether other health behaviours also deteriorate. A stoma has also been identified as a barrier to improving health behaviours [18], highlighting a need for targeted interventions that address stoma-related concerns associated with health behaviours. In particular, previous research has found that patients report a fear of exercise after stoma surgery, with fears of developing a parastomal hernia representing a major barrier for this group [17].

There is a paucity of research exploring lifestyle interventions in people with stoma, but self-management interventions that touch on lifestyle topics such as diet and PA have shown promise [19–21]. For example, an educational intervention that included a session on exercising after stoma creation [22] and another including two training sessions on muscle relaxation and practice at home for ten weeks [23], both reported a positive effect on QOL. Similarly an intervention that included recommendations for abdominal exercises alongside wearing a support garment appeared to find a significant reduction in hernia incidence [24]. However, studies to date have been small and/ or methodologically flawed. Dietary and PA interventions for colorectal cancer survivors provide more robust evidence for positive changes in behaviour, QOL, and overall health [18, 25–27].

However, although some self-management interventions for stoma patients include lifestyle advice, these kinds of interventions have not been implemented in routine stoma patient care pathways. Little is known about whether lifestyle is routinely discussed after a stoma, nor how useful any information received is thought to be. The Association of Stoma Care Nurses UK (ASCN) national guidelines [28] recommend that patients be provided with advice on appropriate abdominal and core muscle exercises after surgery to rehabilitate the abdominal wall, and guidelines from The World Cancer Research Fund recommend that cancer patients should follow their guidelines for the general population with respect to PA, diet, alcohol and smoking [29]. However, a recent survey of people with a stoma found 68% had not been given information about PA at any stage of their surgery or recovery, and 82% had not received advice about core or abdominal exercises, though most would have liked guidance [17]. Gaining a better understanding of the type of advice people receive about lifestyle following stoma formation surgery, how satisfied they were with the information, and if they would be open to receiving lifestyle advice, could help identify specific gaps and inform interventions going forward.

The aim of the survey reported in this article was to describe current lifestyle (PA, diet, alcohol consumption, and smoking) and changes in lifestyle following stoma formation. Furthermore, we wanted to explore associations between concerns about stoma and any reported changes in lifestyle, and to describe the provision of and desire for lifestyle information, advice and support among this group. The purpose of the study was to inform future development of lifestyle interventions for people undergoing stoma formation surgery to improve their overall health and QOL.

## Methods

A cross-sectional survey was conducted in the UK in 2016. Questions to elicit people’s self-reported lifestyle, concerns about permanent stoma and experiences of lifestyle information and advice were included and it was administered on-line using Bristol Survey On-line (BOS). An on-line questionnaire is a well-established method used in health research [30, 31] and was chosen as a practical way of quickly reaching eligible people to complete the survey. The School of Health Sciences, University of Stirling research and ethics committee approved the study.

### Participants and recruitment

People 16 years of age and over who previously or currently had a stoma for treatment for any medical condition were invited to take part. We included individuals who had a stoma in the past, but did not currently have a stoma, as these individuals would still be able to speak to their experiences around the receipt of lifestyle advice at the time of their stoma formation. Relevant UK charities (Colostomy Association, Ileostomy Association and Bowel and Cancer Research) and Coloplast (a company supplying stoma-related appliances) included a link on their web site and on social media (e.g. Facebook) to the study’s on-line questionnaire hosted by BOS. The first web page of the survey provided information about the study. To access the questionnaire, potential participants were required to give a form of written informed consent through ticking a consent box. This is an approach to obtaining consent used in previous on-line health research projects [32], and was approved by the University ethics committee. The questionnaire was in English and the sampling approach and recruitment procedure were chosen for convenience and are commensurate with the purpose, scale and limited resources of the study.

### Variables

Respondents were asked to provide their age and gender and to indicate: what medical condition their first stoma was related to, if they still had a stoma, how long they had been living with a stoma or how long they had a stoma before it was closed, and if they had ever seen a stoma nurse specialist.

Four validated single items were used to determine respondents’ current smoking, diet, PA and alcohol consumption. Current smoking status was assessed using the recommended key question from the key questions from the Global Adult Tobacco Survey (GATS): Do you currently smoke tobacco on a daily basis, less than daily or not at all? [33] Self-rated diet quality was measured on a 5-point Likert scale: In general, how healthy is your overall diet? Would you say [1] excellent [2], very good [3], good [4], fair, or [5] poor? [34]. Participants were also asked to report the number of fruit and vegetable servings eaten the day before (0/ 1–4/ ≥5); a measure that has previously been shown to correlate well with a biomarker of fruit and vegetable intake [35]. Physical activity was measured using the item “In the past week, on how many days have you done a total of 30 minutes or more of physical activity, which was enough to raise your breathing rate. This may include sport, exercise, and brisk walking or cycling for recreation or to get to and from places, but should not include housework or physical activity that may be part of your job” [36]. Alcohol consumption was assessed using the question, “How many times in the past year have you had X or more drinks in a day?” (where X is 5 for men and 4 for women) [37]. An additional four items were used to find out if respondents had changed their lifestyle since having a stoma. For each behaviour, respondents were asked to select from four options; for example, for the item PA, respondents could choose either ‘Yes I do more physical activity’, ‘Yes I do less physical activity’, ‘No change’, ‘Don’t know.’

With respect to concerns about stoma, one item was used to determine any concerns that respondents currently have or have had in the past about the possibility of a permanent stoma. A list of 15 concerns, drawn from empirical evidence about QOL and experiences of living with a stoma [38–40] was presented for respondents to select. These items were drawn from the literature and discussed by the research team (ie. listed authors) with representatives from relevant organisations (the Colostomy Association, Ileostomy Association, Urostomy Association and Bowel and Cancer Research) and a patient advisor, to ensure relevant concerns were included.

Finally, eight items were used to find out if respondents had been given any information, advice or support after their original stoma about PA, diet, smoking and alcohol consumption. For each behaviour, respondents selected from the following four options: ‘Yes’, ‘No and I would not have wanted any information or advice ‘, ‘No and I would have liked to have received some information and advice’, and ‘Don’t know’. If respondents answered ‘Yes’ they were asked if the advice and support was useful and given the following three options: ‘Yes’, ‘No’ and ‘Don’t know.’

The layout and order of the questionnaire was piloted with members of the research team, organisation representatives and the patient advisor, who also confirmed face validity with respect to the study aims. The final questionnaire can be found in Additional file 1.

### Sample size

The minimum sample size was anticipated to be around 400 participants, based on an expected 10% response rate from members of the charities involved. With this sample size, proportions of responses to questions on the survey could be estimated to within +/− 5% (width of 95% confidence interval).

### Analysis

Responses to the survey questions were summarised using descriptive statistics, and compared between groups of participants (e.g. age, gender, medical condition etc.) using chi-squared or Fisher’s exact tests, as appropriate. For those participants who reported concerns about stoma, we also explored descriptively the proportions reporting changes in their health behaviours as an indicator of how these concerns might be associated with lifestyle after stoma.

## Results

### Participants

There were 425 responses in total. The majority were female (76%); the median age group was 41–50 years; the most frequently reported main reason for a first stoma was Inflammatory Bowel Disease (IBD) (39%), followed by colorectal cancer (CRC) (22%), then diverticular disease (DD) (11%); 93% of respondents still had their stoma at the time of completing the survey; the length of time living with stoma ranged from < 6 months to over 5 years (median: 2–3 years); 97.4% had seen a stoma nurse specialist (Table 1).

**Table 1:** Respondent characteristics (*n* = 425)

Characteristics	Respondents (n (%)^a^
Gender	
Male	102 (24.1)
Female	322 (75.9)
Unknown	1
Age when first had stoma	
< 20	26 (6.1)
21–30	44 (10.4)
31–40	83 (19.5)
41–50	98 (23.1)
51–60	89 (20.9)
61–70	64 (15.1)
> 70	21 (4.9)
Reason for first stoma	
Inflammatory Bowel Disease	165 (39.4)
Colorectal cancer	93 (22.2)
Diverticular disease	47 (11.2)
Bowel perforation / rupture	24 (5.7)
Other cancer or benign tumour	20 (4.8)
Other	70 (16.7)
Unknown	6
Stoma at time of survey?	
Yes	397 (93.4)
No	28 (6.6)
Length of time with stoma	
< 1 year	117 (27.6)
1–2 years	73 (17.2)
2–4 years	90 (21.2)
> 5 years	144 (34.0)
Unknown	1
Seen stoma nurse specialist?	
Yes	409 (97.4)
No	11 (2.6)
Unknown	5

^a^ Percentages calculated excluding the unknowns

### Current lifestyle

Table 2 presents the self-reported behaviours of respondents (presented according to medical reason for first stoma in Appendix Table 6). Current levels of PA were low: 30% reported they had not done > 30 min of PA on any day during the previous week; the median number of days of PA was 3 (interquartile range 1–5), with no significant difference according to reason for stoma (*p* = 0.852). Most respondents considered that their diet was healthy (63% reported excellent / very good / good), regardless of reason for stoma (*p* = 0.379), although only 20% overall reported eating at least 5 servings of fruit and vegetables the previous day. Most respondents were non-smokers (84%), with cancer patients less likely to smoke (5% compared with 18% for IBD/DD and 24% for other, *p* = 0.001). Most respondents’ alcohol consumption was in line with the UK National Health Service (NHS) recommendations for keeping intake below 14 units a week (if you drink) [41]. 60% reported *never* having > 8 units (for men) or > 6 units (for women) on one occasion, and 21% reported this less than monthly. Older respondents were less likely to drink more than the specified number of units on one occasion, with 71% of those aged > 50 reporting that they never did this, compared with 53% of those aged < 50 (*p* = 0.016 for trend across categories of alcohol consumption); drinking patterns were similar according to reason for stoma (*p* = 0.138). There were no significant differences in behaviours according to length of time with the stoma or gender.

**Table 2:** Current lifestyle (n = 425)

Behaviours	n (%)^a^
Physical activity	
*Number of days > 30 mins physical activity to raise breathing rate (in the last week)*	
0	127 (29.9)
1	49 (11.5)
2	62 (14.6)
3	67 (15.8)
4	26 (6.1)
5	38 (9.0)
6	12 (2.8)
7	43 (10.1)
Unknown	1
*Median (interquartile range)*	*3 (1–5)*
Diet	
*In general, how healthy is your overall diet now?*	
Excellent	18 (4.2)
Very good	90 (21.3)
Good	157 (37.1)
Fair	110 (26.0)
Poor	48 (11.3)
Unknown	2
*Number of fruit and vegetable servings eaten the day before*	
None	55 (13.1)
1–4	280 (66.7)
> 5	85 (20.2)
Unknown	5
Smoking	
*Currently smoke tobacco*	
Daily	55 (13.0)
Less than daily	13 (3.1)
Not at all	354 (83.9)
Unknown	3
Alcohol	
*How often have > 8 (men) or > 6 (women) standard drinks (units) on one occasion*	
Never	250 (60.5)
< Monthly	88 (21.3)
Monthly	34 (8.2)
Weekly	33 (8.0)
Daily or almost daily	8 (1.9)
Unknown	12

^a^ Percentages calculated excluding the unknowns

### Change in lifestyle after a stoma

Table 3 describes the proportion of respondents reporting a change in lifestyle since having a stoma (presented according to medical reason for first stoma in Appendix Table 7). 56% reported that they had decreased the amount of PA that they did since having a stoma, with only 15% increasing their levels of PA and 27% reporting no change. In contrast, respondents were more likely to report improvements in diet and alcohol consumption since having a stoma, with 36% reporting a healthier diet (23% a less healthy diet) and 34% drinking less alcohol.

**Table 3:** Change in lifestyle since having a stoma (n = 425)

	PA n (%)^a^	Diet n (%)^a^	Alcohol n (%)^a^	Smoking n (%)^a^
For the better	64 (15.1)	153 (36.3)	146 (34.6)	52 (12.6)
For the worse	238 (56.1)	99 (23.5)	20 (4.7)	11 (2.7)
No change	113 (26.6)	159 (37.8)	251 (59.5)	344 (83.5)
Don’t know	9 (2.1)	10 (2.4)	5 (1.2)	5 (1.2)
Missing	1	4	3	13

^a^ Percentages calculated excluding the missing responses

Change in levels of PA differed significantly according to medical reason for the first stoma (*p* = 0.025) with IBD/DD patients more likely to increase PA (18% for IBD/DD compared with 12% for cancer) and less likely to reduce PA (49% for IBD/DD compared with 64% for cancer) (Appendix Table 7).

Changes in levels of PA also differed significantly by age. Respondents in the oldest age group (> 70) were more likely to report doing less PA since their stoma (76%) and those in the youngest age group (< 20) more likely to report doing more PA (35%) (*p* = 0.036 for overall comparison of all age groups). Older respondents (aged > 50) were more likely to report a healthier diet since their stoma (45% compared with 32% of those aged < 50, *p* = 0.024).

### Concerns about permanent stoma and lifestyle

Frequencies of concerns about a permanent stoma were high. The 10 most commonly reported concerns were: pouch loosening and leaking (69%), smell and odour (60%), feeling unattractive/different (59%), energy levels (55%), skin problems (54%), tiredness (51%), sex life affected (49%), impact on social life (44%), pain and feeling uncomfortable around stoma site (42%), bowel function problems (37%).

Respondents reporting concerns about a permanent stoma were more likely to report that their levels of PA had reduced since having the stoma (Table 4). This was particularly true for individuals concerned about energy levels, feeling very tired, the impact of the stoma on their social life, pain/discomfort around stoma site and elsewhere in their body, getting back to normal activities, and nausea.

**Table 4:** Concerns about a permanent stoma according to change in physical activity since stoma

Concern	Change in PA since stoma			Total
	More	Less	No change	
The pouch loosening and leaking	44 (15%)	170 (59%)	72 (25%)	280
Smell and odour	38 (15%)	155 (63%)	53 (22%)	246
Feeling unattractive/different	41 (17%)	158 (66%)	42 (17%)	241
My energy levels	27 (12%)	167 (73%)	35 (15%)	229
Skin problems	31 (14%)	142 (64%)	50 (22%)	223
Being very tired	24 (11%)	152 (72%)	35 (17%)	211
Sex life being affected	35 (17%)	130 (64%)	39 (19%)	204
Impact on my social life	22 (12%)	135 (75%)	24 (13%)	181
Pain and feeling uncomfortable about stoma site	24 (14%)	119 (70%)	27 (16%)	170
Bowel function problems (constipation, diarrhoea)	20 (13%)	104 (68%)	28 (18%)	152
Getting back to normal activities around the house	20 (13%)	108 (71%)	24 (16%)	152
Pain in other regions of my body (e.g. back)	12 (9%)	99 (77%)	18 (14%)	129
Getting back to work	20 (15%)	86 (67%)	23 (18%)	129
Effect on my family	25 (21%)	79 (68%)	13 (11%)	117
Nausea	5 (9%)	48 (81%)	6 (10%)	59
Any other concerns	3 (8%)	26 (65%)	11 (27%)	40

There were no notable differences in those reporting a change in diet since having a stoma according to specific concerns, with the exception that 40% of those concerned about nausea reported a less healthy diet since the stoma. The numbers in the categories for change in smoking and drinking habits since the stoma were too small for this analysis.

### Lifestyle information, advice and support after a stoma

51, 67, 25 and 19% of respondents reported that they had received information, advice or support about PA, diet, alcohol and smoking, respectively (Table 5). Of those that had received advice, the overwhelming majority reported that it was useful; this ranged from 78% for smoking to 92% for PA and alcohol.

**Table 5:** Information, advice or support received about lifestyle behaviours (n = 425)

	PA n (%)	Diet n (%)	Alcohol n (%)	Smoking n (%)^a^
After your original stoma surgery, were you ever given any information, advice or support about the following:				
Yes	219 (51.5)	283 (66.6)	105 (24.7)	81 (19.2)
No and I would not have wanted any information or advice	14 (3.3)	12 (2.8)	156 (36.7)	246 (58.3)
No and I would have liked to have received some information and advice	177 (41.6)	127 (29.9)	135 (31.8)	49 (11.6)
Don’t know	15 (3.5)	3 (0.7)	29 (6.8)	46 (10.9)
Missing				3
If yes, was the advice and support useful?				
Yes	202 (92.2)	233 (82.3)	97 (92.4)	63 (77.8)
No	11 (5.0)	41 (14.5)	6 (5.7)	15 (18.5)
Don’t know	6 (2.7)	9 (3.2)	2 (1.9)	3 (3.7)

^a^ Percentages calculated excluding the missing responses

42% reported that they had not received any information, advice or support on physical activity but would have welcomed it. 30, 32 and 12% reported that they had not received information, advice or support on diet, alcohol and smoking, respectively but would have welcomed it.

Some respondents reported that they would not have wanted lifestyle information, advice and support, though this may reflect that certain kinds of advice were not relevant for all participants. For example, of the 156 who said that they would not have wanted information, advice or support about alcohol, 114 (73%) were in the lowest category of reported alcohol consumption (never drank more than the specified number of units on one occasion) and of the 246 who said that they would not have wanted information, advice or support about smoking, 225 (91%) were currently non-smokers.

## Discussion

In this study, people who previously or currently had a stoma were generally non-smokers, and most were meeting recommendations for alcohol. However, only 1/5 reported eating at least 5 servings of fruit and vegetables the previous day, and nearly 1/3 had not engaged in any physical activity during the previous week. Most felt their PA had decreased following stoma formation, and nearly ¼ reported that their diet had worsened. Frequencies of concerns about a permanent stoma were high, and associated with the reported decreases in PA. Of those reporting nausea, 40% felt their diet had worsened. While the majority of people recalled receiving useful lifestyle advice, a large proportion had not received PA (42%) or diet (30%) advice, and would have welcomed it.

This is one of the first studies to explicitly explore lifestyle in people who have had stoma formation surgery. Our finding that PA had decreased for most people, is in line with previous survey research exploring PA in people with a stoma [18]. Studies conducted among people with diseases associated with stoma surgery have also found decreases in PA among people with colorectal cancer [42]^2^ and inflammatory bowel disease [43]. Hence, a decrease in PA may be related to disease rather than the stoma per se. However one study has shown that among colorectal cancer patients, decreases in PA are greater for those patients with a stoma [16]. Similarly, studies reporting barriers to being physically active among people with colorectal cancer suggest that a stoma may be a deterrent [18, 44]. In our study decreases in PA were associated with a number of stoma-related concerns, including pain/discomfort around stoma site. Prospective studies are needed to determine whether these concerns lead to reported decreases in PA, and interventions seeking to increase PA within this group may need to find ways to address these issues.

To our knowledge, no other published studies have directly examined other lifestyle behaviours in people who have had stoma formation surgery. Although most respondents in our sample were meeting the NHS recommendations for alcohol intake, and few reported smoking, only a small percentage were meeting the daily recommendations for fruit and vegetable intake. Furthermore, a number of patients reported that their diet had become less healthy post-stoma surgery, and nausea appeared to be related to a decrease in diet quality. Again, prospective studies are needed to explore this in more depth, and utilizing more detailed measures of dietary intake. However, these results suggest that for some patients, dietary support may be as important as PA advice, and indeed almost 1/3 of respondents felt they would benefit from this.

There has been little research on the provision of lifestyle advice after stoma formation surgery. A higher proportion of our survey respondents reported receiving PA advice compared to the previous survey, which found that 2/3rds of respondents with a stoma had not received this advice [18]. This may reflect differences in our samples; respondents to our survey were younger and most had surgery for IBD, whereas in the previous survey the main reason for stoma surgery was ulcerative colitis. However, it is still of concern that half of our participants had not received any advice, despite recommendations suggesting this should be provided as part of their care [29]. The results from this study also suggests that lifestyle information, advice and support vary; more respondents received information, advice and support about diet than about PA. It is encouraging that those who had received advice reported finding it useful. Previous studies with both cancer patients, and people with a stoma suggest that those who receive advice are more likely to engage in the recommended behaviour [18, 45]. Given that most people with a stoma would welcome guidance, supporting health professionals to provide this could improve their patients’ behaviour, care, and ultimately quality of life.

This study has a number of limitations, including use of convenience sampling and in particular, recruitment bias, which limits the external validity of our findings. Respondents recruited via charity web sites are unlikely to be typical or representative of the total population of people with a stoma, and we are unable to determine the number of eligible participants who were able to access our questionnaire but chose not to participate. Nonetheless, this study gives some evidence about the provision of lifestyle information and advice and whether some people who have a stoma (i.e. those who access charity information and support on-line) are receptive to lifestyle advice. The survey was restricted to online users only, who may be particularly motivated to seek information and guidance, and may have higher internet literacy. There is also the chance that users could complete the survey more than once and influence the results. The study relied on self-report, which again may bias findings.

The study sample was heterogeneous in terms of age and reason for first stoma, and group differences were observed. Older patients were more likely to report a healthier diet, whereas younger patients, and those who had a stoma because of IBD/DD were less likely to have reduced their PA. Younger respondents are also more likely to have had IBD, which makes it difficult to determine if the observed differences are a consequence of age or disease, or both. Patients with IBD/DD may also have been managing bowel complaints for a longer period of time prior to stoma formation. Our study did not ask about stoma type, which may also impact lifestyle and lifestyle change. We included participants who had a stoma reversal as well as those who currently had a stoma. Only 6.6% of respondents did not have a stoma at the time of completion so we were unable to compare groups, but the impact of a stoma reversal on lifestyle may differ. Future research should explore whether lifestyle interventions need to be tailored to different clinical subgroups. Additionally, although we found no differences by gender or time since stoma, this may reflect the fact that the majority of our sample were female, and median time since stoma was 2–3 years. Studies with larger, more diverse samples should explore whether there are different needs among patients who have had a stoma for longer, and among men.

## Conclusions

Few respondents to this survey were eating the recommended amount of fruit and vegetables, and most reported a decrease in their PA following stoma surgery. Importantly, a higher proportion of those reporting concerns about their permanent stoma reported decreases in PA. People reported varied experiences in terms of receiving lifestyle advice after stoma surgery, but most would welcome guidance. Developing interventions that support health professionals to provide evidence-based lifestyle advice, while also addressing patients’ concerns about their stoma, could help patients to change their health behaviours and improve satisfaction with care.

### Additional file

Additional file 1: Stoma and lifestyle questionnaire. (PDF 171 kb)

## Appendix

**Table 6:** Current lifestyle, according to medical reason for first stoma (*n* = 419^a^)

Behaviours	Chronic bowel diseasen (%)^b, c^	Cancer^c^ n (%)^b, d^	Other n (%)^b^
Physical activity			
*Number of days > 30 mins physical activity to raise breathing rate (in the last week)*			
0	60 (28.4)	36 (31.9)	28 (29.8)
1	24 (11.4)	13 (11.5)	12 (12.8)
2	37 (17.5)	8 (7.1)	17 (18.1)
3	33 (15.6)	20 (17.7)	14 (14.9)
4	15 (7.1)	6 (5.3)	5 (5.3)
5	16 (7.6)	16 (14.2)	5 (5.3)
6	5 (2.4)	3 (2.6)	3 (3.2)
7	21 (10.0)	11 (9.7)	10 (10.6)
Unknown	1	0	0
*Median (interquartile range)*	*3 (1–5)*	*3 (1–6)*	*3 (1–4)*
Diet			
*In general, how healthy is your overall diet now?*			
Excellent	8 (3.8)	5 (4.5)	5 (5.4)
Very good	48 (22.6)	27 (24.1)	15 (16.1)
Good	74 (34.9)	46 (41.1)	35 (37.6)
Fair	56 (26.4)	28 (25.0)	23 (24.7)
Poor	26 (12.3)	6 (5.4)	15 (16.1)
Unknown	0	1	1
*Number of fruit and vegetable servings eaten the day before*			
None	33 (15.7)	8 (7.1)	11 (12.0)
1–4	137 (65.2)	77 (68.8)	63 (68.5)
> 5	40 (19.1)	27 (24.1)	18 (19.6)
Unknown	2	1	2
Smoking			
*Currently smoke tobacco*			
Daily	28 (13.3)	6 (5.4)	20 (21.5)
Less than daily	11 (5.2)	0	2 (2.2)
Not at all	172 (81.5)	106 (94.6)	71 (76.3)
Unknown	1	1	1
Alcohol			
*How often have > 8 (men) or > 6 (women) standard drinks (units) on one occasion*			
Never	120 (58.2)	72 (66.1)	55 (59.1)
< Monthly	47 (22.8)	15 (13.8)	26 (28.0)
Monthly	19 (9.2)	12 (11.0)	2 (2.2)
Weekly	15 (7.3)	9 (8.3)	8 (8.6)
Daily or almost daily	5 (2.4)	1 (0.9)	2 (2.2)
Unknown	6	4	1

^a^ Medical reason for first stoma was unknown for 6 participants. ^b^ Percentages calculated excluding the unknowns; ^c^ Includes Inflammatory bowel disease and diverticular disease; ^d^ Includes colorectal cancer, other cancer types and benign tumours

**Table 7:** Change in lifestyle since having a stoma, according to medical reason for first stoma (n = 419^a^)

Change in lifestyle	PA n (%)^b^			Diet n (%)^b^			Alcohol n (%)^b^			Smoking n (%)^b^		
	Chronic bowel disease^c^	Cancer^d^	Other	Chronic bowel disease^c^	Cancer^d^	Other	Chronic bowel disease^c^	Cancer^d^	Other	Chronic bowel disease^c^	Cancer^d^	Other
For the better	37 (17.5)	14 (12.4)	13 (13.8)	71 (34.1)	48 (42.5)	33 (35.1)	74 (35.1)	42 (37.5)	29 (31.2)	24 (11.6)	16 (14.7)	9 (10.0)
For the worse	104 (49.3)	72 (63.7)	58 (61.7)	50 (24.0)	25 (22.1)	23 (24.5)	13 (6.2)	3 (2.7)	4 (4.3)	6 (2.9)	0	5 (5.6)
No change	64 (30.3)	26 (23.0)	22 (23.4)	83 (39.9)	37 (32.7)	36 (38.3)	122 (57.8)	65 (58.0)	60 (64.5)	176 (85.0)	89 (81.6)	76 (84.4)
Don’t know	6 (2.8)	1 (0.9)	1 (1.1)	4 (1.9)	3 (2.6)	2 (2.1)	2 (0.9)	2 (1.8)	0	1 (0.5)	4 (3.7)	0
Missing	1	0	0	4	0	0	1	1	1	5	4	4

^a^Medical reason for first stoma was unknown for 6 participants; ^b^ Percentages calculated excluding the missing responses; ^c^ Includes Inflammatory bowel disease and diverticular disease; ^d^ Includes colorectal cancer, other cancer types and benign tumours

## Abbreviations

ASCN: Association of Stoma Care Nurses UK
BOS: Bristol Online Survey
CRC: colorectal cancer
DD: diverticular disease
GATS: Global Adult Tobacco Survey
IBD: Inflammatory Bowel Disease
NHS: National Health Service
PA: Physical Activity
QOL: Quality of Life
UK: United Kingdom
