# Supplementary material for: Smoking, alcohol consumption, diet and physical activity following stoma formation surgery, stoma-related concerns, and desire for lifestyle advice: a United Kingdom survey
Source: BMC Public Health. 2019 May 15;19:574. doi: 10.1186/s12889-019-6913-z (PMC6518630; doi:10.1186/s12889-019-6913-z)
Supplement: Supplementary file 1 — Stoma and lifestyle questionnaire. (PDF 171 kb) [file 12889_2019_6913_MOESM1_ESM.pdf]

Stoma (copy) (copy)

Page 1

You are invited to take part in this survey being conducted by researchers at the University of Stirling.

What is the purpose of the study?

We are interested in learning more about the lifestyles of people who have had a stoma. The main purpose of this survey is to find out if people receive lifestyle information after having a stoma, and to find out if people who have a stoma would be interested in a lifestyle intervention.

Who should complete this questionnaire?

It is up to you to decide if you want to take part.

We would like anyone over 16 years of age who has had or currently has a stoma, including a colostomy and iliostomy, to be involved in the survey.

What will it involve?

We would like you to complete a questionnaire, which may take up to 20 minutes to complete.

Advantages and Disadvantages about taking part?

This study aims to help people who have a stoma in the future. Some people may find that taking part in this study brings back memories about their illness and become upset. If you do become upset then please contact one of the charities that are supporting this study for help and advice. Their contact details can be found below.

What will happen at the end of the study?

At the end of the study the charities involved in this survey will be sent a summary of the results that they will post on their website. There will be no

further contact with you by the research team.

What about confidentiality?

We will not ask for your name or your address. You will not therefore be named in any results or reports written about the study. All data can only be read by researchers. Data will be kept in a locked filing cabinet and in password protected folders on computers at the University of Stirling and will be destroyed in 10 years time.

Ethical Approval

This study has approval from the University of Stirling.

Who do I contact for further information about the study?

Dr Gill Hubbard is leading this study. She can be contacted at the following address or by email:

Dr Gill Hubbard, School of Health Sciences, University of Stirling, Highland Campus, Centre for Health Science, Old Perth Road, Inverness, IV2 3JH.

Email: gill.hubbard@stir.ac.uk

The charities that we are working with are:

Colostomy association

http://www.colostomyassociation.org.uk/

Stoma wise

http://www.stomawise.co.uk/

Ileostomy association

http://www.iasupport.org/

What if I wish to complain about the study?

You can submit a written complaint about the study to: Dr Annetta Smith, School of Health Sciences, University of Stirling, Highland campus, Centre for Health Science, Old Perth Road, Inverness, IV2 3JH.

Or you can contact her by telephone on: 01463 255646

Or you can email her at: Annetta.Smith@stir.ac.uk

Page 2

1 I consent to take part in this survey ✱ *Required*

- ○ Yes

Page 3

2 What is your gender? For example, male, female. ✱ *Required*

Page 4

3 What age were you when you first had a stoma? Please select. ✱ *Required*

- 15 years or under
- 16 - 20 years
- 21 - 30
- 31 - 40
- 41 - 50
- 51 - 60
- 61 - 70
- 71 - 80
- Over 80

Page 5

4 Please tick if your first stoma was related to treatment for: ✱ *Required*

- ☐ Rectal cancer
- ☐ Colon cancer
- ☐ Irritable bowel syndrome
- ☐ Inflammatory bowel disease
- ☐ Diverticular disease
- ☐ Crohn's disease
- ☐ Ulcerative colitis
- ☐ Other
- ☐ Don't know

4.a If you selected Other, please specify:

Page 6

5 Do you still have a stoma? Please select the relevant option ✱ *Required*

Page 7

6 Please select how long you have been living with a stoma, or how long you had a stoma before it was closed.

- 0 - 6 months
- 7 - 12 months
- 13 - 18 months
- 19 - 24 months
- 25 - 36 months
- 37 - 48 months
- More than 5 years

Page 8

7 Have you ever seen a stoma nurse specialist? Please select appropriate response.

- Yes
- No
- Don't know

Page 9

8 After your original stoma surgery, were you ever given any information, advice or support about any of the following?

Physical activity

- ◯ Yes
- ◯ No and I would not have wanted any information or advice about physical activity
- ◯ No and I would have liked to have received some information and advice about physical activity
- ◯ Don't know

8.a Was this advice and support useful?

- ☐ Yes
- ☐ No
- ☐ Don't know

9 Diet

- ◯ Yes
- ◯ No and I would not have wanted any information or advice about diet
- ◯ No and I would have liked to have received some information or advice about diet
- ◯ Don't know

9.a Was this advice and support useful?

- ◯ Yes
- ◯ No

- Don't know

10 Smoking/ Tobacco

- Yes
- No and I would not have wanted any information or advice about smoking cessation
- No and I would have liked to have received some information and advice about smoking cessation
- Don't know

10.a Was this advice and support useful?

- Yes
- No
- Don't know

11 Alcohol consumption

- Yes
- No and I would not have wanted any information or advice about alcohol consumption
- No and I would have liked to have received some information and advice about alcohol consumption
- Don't know

11.a Was this advice and support useful?

- Yes

- No
- Don't know

Page 10

12 Please tell us any concerns *you currently have or have had in the past* about the possibility of a permanent stoma. You can select as many as appropriate.

- ☐ Pain and feeling uncomfortable about the stoma site
- ☐ Being very tired
- ☐ My sex life being affected
- ☐ Feeling unattractive / different
- ☐ Bowel function problems (e.g. constipation, diarrhoea)
- ☐ Nausea
- ☐ Impact on my social life
- ☐ Smell and odour
- ☐ Effect on my family
- ☐ The pouch loosening and leaking
- ☐ Skin problems
- ☐ Pain in other regions of my body (e.g. Back)
- ☐ Getting back to normal activities around the house
- ☐ Getting back to work
- ☐ My energy levels
- ☐ Any other concerns
- ☐ I have been told my stoma will definitely be reversed

12.a We may not have listed all your concerns. Please include any other concerns you have or have had regarding a permanent stoma here

13 Please tell us any concerns *you currently have or have had* in the past about stoma reversal and closure.

- ☐ Potential problems after the operation such as wound infections
- ☐ That the surgeons won't be able to reverse it
- ☐ Length of hospital stay
- ☐ Incontinence
- ☐ Pain and feeling uncomfortable
- ☐ Being very tired
- ☐ My sex life being affected
- ☐ Feeling unattractive
- ☐ Bowel function (e.g. constipation or diarrhoea)
- ☐ Nausea
- ☐ The impact of my social life
- ☐ Smell and odour
- ☐ The effect on my family
- ☐ Risk of dying through the surgery
- ☐ Fear of the cancer returning
- ☐ Length of time having to wait on the reversal
- ☐ Any other concerns
- ☐ I have no concerns

13.a We may not have included all the issues that concern you. Please add any other concerns you have or have had regarding stoma reversal and closure here

Page 12

14 Have you changed your lifestyle since having the stoma?

Level of physical activity

- ◯ Yes I do more physical activity
- ◯ Yes I do less physical activity
- ◯ No change
- ◯ Don't know

15 Diet

- ◯ Yes I eat a healthier diet
- ◯ Yes I eat a less healthy diet
- ◯ No change
- ◯ Don't know

16 Smoking and Tobacco

- ◯ Yes I stopped smoking
- ◯ Yes I started smoking
- ◯ No change
- ◯ Don't know

17 Alcohol consumption

- ◯ Yes I drink less alcohol

- Yes I drink more alcohol
- No change
- Don't know

Page 13

18 In the last week on how many days have you done a total of 30 minutes or more of physical activity, which was enough to raise your breathing rate. This may include sport, exercise, brisk walking or cycling for recreational purposes or to get to and from places, but should not include housework, or physical activity that may be part of your job. Please select one

- 0 days
- 1 day
- 2 days
- 3 days
- 4 days
- 5 days
- 6 days
- 7 days

Page 14

19 In general, how healthy is your overall diet now? Please select

- Excellent
- Very good
- Good
- Fair
- Poor

Page 15

20 Thinking about nutrition how many total servings of fruit and/or vegetables did you eat yesterday? A serving would equal 1 medium apple, a handful of broccoli, or 3 heaped tablespoons of carrots. Please write your total here

Page 16

21 Do you currently smoke tobacco on a daily basis?

- Daily
- Less than daily
- Not at all

Page 17

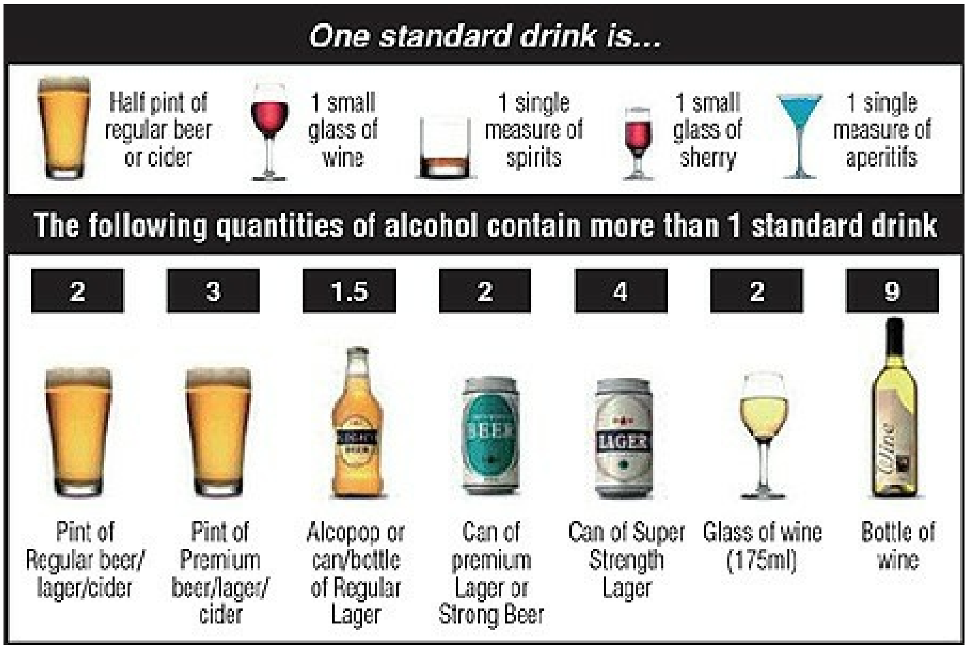

22 Men: How often do you have EIGHT or more standard drinks on one occasion? Women: How often do you have SIX or more standard drinks on one occasion?

- Never
- Less than Monthly
- Monthly
- Weekly
- Daily or almost daily

Page 18

23 If you have any further comments please add them here.

Page 19

Thank you for taking part in this survey. If you have any further questions please contact Dr Gill Hubbard via email on gill.hubbard@stir.ac.uk

If you have any questions about stoma support and advice please contact one of the following charities:

Colostomy association

http://www.colostomyassociation.org.uk/

Stomawise

http://www.stomawise.co.uk/

Ileostomy association

http://www.iasupport.org/

Key for selection options

5 - Do you still have a stoma? Please select the relevant option

Yes
No
